# What’s Past Is Prologue: History of Nonalcoholic Fatty Liver Disease

**DOI:** 10.3390/metabo10100397

**Published:** 2020-10-08

**Authors:** Giovanni Targher

**Affiliations:** Department of Medicine, Section of Endocrinology, Diabetes and Metabolism, University of Verona, 37126 Verona, Italy; giovanni.targher@univr.it; Tel.: +39-045-8123748

**Keywords:** NAFLD, nonalcoholic fatty liver disease, MAFLD, metabolic dysfunction-associated fatty liver disease

## Abstract

Since the initial descriptions in the early 1980s by Dr. Ludwig et al. and Drs. Schaffner and Thaler, who firstly coined the terms nonalcoholic steatohepatitis (NASH) and nonalcoholic fatty liver disease (NAFLD), this liver disease has become a global health problem worldwide, causing considerable liver-related and extra-hepatic morbidity and mortality. Based on pathophysiological insights gained from the past decades, it has been clearly established that NAFLD is a metabolic liver disease whose etiology and pathogenesis extends beyond the liver and that NAFLD has important clinical implications, especially in terms of an increased risk of developing both cardiovascular disease (which represents the leading cause of death in this patient population) and other extra-hepatic manifestations, such as type 2 diabetes mellitus, chronic kidney disease, and some extra-hepatic cancers. The aim of this brief commentary is to discuss a recent review article written by Dr. Lonardo and colleagues, who raised awareness of the history of NAFLD. Since “What’s past is prologue”, I believe that this review article focusing on the history of NAFLD may contribute to better understanding the disease itself, as well as to anticipating the lines of the future clinical and pharmacological research of this common and burdensome liver disease.

“What’s past is prologue” is a quotation by William Shakespeare from his play The Tempest that is also engraved on the marble statue, The Future, located at the National Archives Building in Washington, DC. 

Keeping this Shakespeare quotation in mind, I believe that Dr. Lonardo and colleagues are to be congratulated for their effort(s) to raise awareness of the history of nonalcoholic fatty liver disease (NAFLD) [1]. Indeed, in their narrative review article, the authors have elegantly summarized the principal historical steps in the study of NAFLD, both in adults and in children, by identifying four major areas of research interest, namely histopathology; clinical correlates–natural course; guidelines issued by scientific societies; and cellular and molecular pathophysiology of NAFLD [1].

Over the last two decades, NAFLD has reached epidemic proportions and is now recognized as a public health problem that affects up to nearly a third of the world’s adult population [2,3]. The burden of NAFLD is strongly influenced by the global epidemics of obesity and type 2 diabetes mellitus, and the prevalence of these conditions is expected to dramatically increase in the forthcoming decades. Thus, NAFLD is an important cause of a poor quality of life for many patients and results in a considerable global health and economic burden for healthcare providers [3,4].

Since the initial descriptions in the early 1980s by Dr. Ludwig et al. [5] and Dr. Schaffner et al. [6], who firstly coined the terms nonalcoholic steatohepatitis (NASH) and NAFLD, respectively, the number of published papers on NAFLD has increased exponentially over time, principally over the last decade.

Figure 1 shows the timeline of publications (number of published papers per year) from the PubMed website using the keyword “nonalcoholic fatty liver disease”, with a total of nearly 23,800 articles published up to 29 September 2020. Looking at this timeline of publications carefully, it appears that the number of published papers per year on NAFLD has remained very low for over two decades, with much fewer than 100 papers published per year until about the mid-2000s, and then began to grow exponentially starting in 2010–2011, with nearly 1000 papers published per year. Notably, 3561 papers were published on PubMed in 2019 and over 4000 papers are expected to be published in 2020.

Personally, I believe that the growing scientific interest in NAFLD, with its consensual exponential growth in scientific publications since 2010–2011 is, in large part, due to the ever-increasing awareness that NAFLD is a metabolic liver disease (whose etiology and pathogenesis extends beyond the liver) that is not only associated with an increased risk of developing liver-related complications but also has important clinical implications, especially in terms of increased risk of developing cardiovascular disease (which represents the leading cause of mortality in people with NAFLD) and other extra-hepatic complications, such as type 2 diabetes mellitus, chronic kidney disease, and some extra-hepatic cancers (e.g., colorectal cancers) [7,8,9,10,11,12]. In other words, the increasing recognition of NAFLD as a “multisystem” disease [13] has allowed this liver disease to move outside of strict hepatological interest, making it a disease of interdisciplinary medical interest. 

For many years, NAFLD has been considered as a consequence of the metabolic syndrome. However, as reported in the review article by Dr. Lonardo et al. [1], the pathophysiological links between NAFLD and components of the metabolic syndrome (especially type 2 diabetes mellitus) are more complex than previously thought [9,14]. Indeed, the deleterious effects of NAFLD extend far beyond the liver, with an accumulating body of clinical evidence now supporting the notion that NAFLD may precede and/or promote the development of cardiovascular disease and type 2 diabetes mellitus [15]. Furthermore, it has been demonstrated that the risk of developing these cardio-metabolic complications parallels the underlying severity of NAFLD, and especially the severity of liver fibrosis [4,15,16,17].

Based on pathophysiological insights gained from the past decades and to further emphasize the strong link between NAFLD and cardio-metabolic diseases, an international panel of experts has recently proposed a new name and definition for NAFLD in adults, i.e., metabolic dysfunction-associated fatty liver disease (MAFLD) [18]. As also reported in the review article by Dr. Lonardo et al. [1], the proposal to change the terminology from NAFLD to MAFLD is still under intense discussion [19,20,21]. However, this proposed change in terminology is largely influenced by the strong association of this common and burdensome liver disease with type 2 diabetes mellitus and underlying metabolic dysfunction (mainly insulin resistance and visceral adiposity). Personally, I believe that the proposed change of the name from NAFLD to MAFLD holds promise to aid in increasing awareness of this liver disease among both primary care physicians and patients, and decreasing its possible social stigma due to its link to alcohol consumption [19]. Moreover, this newly proposed definition might also promote the establishment of MAFLD clinics run jointly by diabetologists and hepatologists to further improve patient care. To date, however, there remain a number of challenges and uncertainties before making this change [19,20]. That said, further research is required to better establish whether, and how, the proposed change of terminology from NAFLD to MAFLD may impact the risk of developing liver-related complications (advanced fibrosis, cirrhosis, and hepatocellular carcinoma), as well as cardiovascular disease or other extra-hepatic diseases associated with NAFLD [19].

Another more recent cause of the exponential growth in scientific publications on NAFLD is the fact that the increasing global prevalence of this metabolic liver disease has fostered the development of entirely new drug classes. Indeed, several phase-2 and phase-3 randomized controlled trials have been published over the last 10 years or are currently underway, with a variety of promising candidates for treatment of NAFLD (such as insulin sensitizers, modulators of bile acid and energy metabolism, antifibrotic agents, or modulators of lipid metabolism) [22]. It is reasonable to hypothesize that the ideal therapeutic agent(s) in NAFLD would not only ameliorate liver disease but also treat and prevent cardio-metabolic diseases associated with NAFLD [23,24]. It is also reasonable to assume that multiple classes of agents targeting different mechanistic pathways will be needed, because no single agent is likely to control all pathophysiological aspects of this complex liver disease [25].

Although a large body of research has taken place over three decades and has resulted in significant improvements in patient care, many knowledge gaps related to NAFLD still remain. Specifically, and relevant to the review article of Dr. Lonardo et al. [1], the pathogenesis and natural history of NAFLD need better clarification. Understanding why there is substantial inter-individual variation in the risk of both NAFLD progression (i.e., only a proportion of NAFLD patients progress to NASH, cirrhosis, or hepatocellular carcinoma) and extra-hepatic clinical outcomes is clinically important to target diagnostic strategies for “at high risk” individuals [4]. In addition, it is known that there is currently a substantial under-appreciation of NAFLD by both primary care clinicians and patients, possibly due to the asymptomatic phenotype of early and compensated disease stages, the lack of clear and easy diagnostic pathways, and a benign course in many patients affected by NAFLD [26,27]. Thus, future global awareness programs are required for NAFLD and its associated hepatic and extra-hepatic complications. Only with better awareness is it likely that clinicians will seek to diagnose the different stages of this liver disease and be suspicious about potential NAFLD-related extra-hepatic complications [4].

In conclusion, if, as written by William Shakespeare, “What’s past is prologue”, I believe that the recent review article written by Dr. Lonardo and colleagues focusing on the history of NAFLD may contribute to a better understanding of the disease itself and its complex pathophysiological mechanisms, as well as to anticipating the lines of the future (clinical/experimental and pharmacological) research of NAFLD. This is because the farther backward we can look, the farther forward we are likely to see.

## Figures and Tables

**Figure 1:**
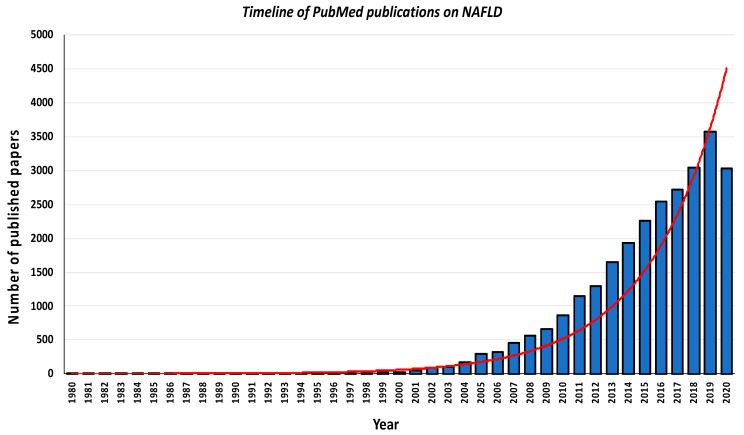
Timeline of published papers (number per year) from the PubMed website using the key “nonalcoholic fatty liver disease” (updated on 29 September 2020). The red line indicates the exponential growth of nonalcoholic fatty liver disease (NAFLD)-related publications over the last 40 years. The blue columns indicate the number of published papers for each year (reported on the *x*-axis).
